# Role of Public-Private Partnerships in Tackling the Tobacco and Obesity Epidemics

**DOI:** 10.5888/pcd11.140134

**Published:** 2014-06-12

**Authors:** Anand K. Parekh, Alicia Richmond Scott, Catherine McMahon, Calvin Teel

**Affiliations:** Author Affiliations: Alicia Richmond Scott, Catherine McMahon, Calvin Teel, US Department of Health and Human Services, Washington, DC.

## Abstract

In response to the illness and death caused by preventable chronic diseases, the US Department of Health and Human Services created Communities Putting Prevention to Work to support community efforts in tackling tobacco use and obesity through policy, systems, and environmental change. As part of this program, 10 national nonprofit organizations with prevention expertise were funded and matched with specific community objectives. Most tobacco and obesity-related matched objectives were successfully accomplished by communities. Public–private partnerships should be considered when addressing chronic disease prevention.

## Objective

In 2009, the American Recovery and Reinvestment Act (ARRA) appropriated $650 million in funding to the US Department of Health and Human Services (HHS) to implement proven prevention and wellness strategies to create a healthier nation. Collaboratively, the Centers for Disease Control and Prevention (CDC) and the Office of the Assistant Secretary for Health developed a new program titled Communities Putting Prevention to Work (CPPW) to 1) increase levels of physical activity, improve nutrition, and decrease obesity rates, and 2) decrease smoking prevalence, teen smoking initiation, and exposure to secondhand smoke. The initiative emphasized policy, systems, and environmental change and provided state and community funding to implement evidence-based interventions for tobacco use control and obesity prevention. Specifically, CDC funded 50 communities proportionate to population size and to the goals of proposed community objectives. The grants ranged from $1 million for small communities to more than $30 million for large communities (1).

A critical component of CPPW included funding 10 national nonprofit organizations at approximately $1 million each for a 24-month period (July 2010–July 2012) to provide technical assistance and guidance to CPPW-funded communities in implementing various public health interventions. National organizations can play a critical role in community disease prevention and health promotion (2–4). Eligible applicants were public or private nonprofit organizations with an established (2 years or longer) national outreach infrastructure, a documented history of health outcomes change resulting from previous projects or campaigns related to tobacco control or obesity prevention, and a national reach that included established networks or affiliates in 5 or more of the 10 HHS regions. Through a competitive process, national organizations were selected on the basis of the following criteria: work plan, organizational capacity and structure, staffing and management plan, evaluation plan, sustainability, and budget narrative.

## Methods

Each national organization was matched with a range of 2 to 12 CPPW-funded communities. Community awardees were typically local or state health departments. The matching process was based on the specific expertise provided by each national organization and the type of support needed by each community to achieve its objectives. The design of this initiative attempted to ensure that every community that could be directly supported by a national organization was matched.

National organization activities included providing mentorship and technical assistance to communities, leveraging financial or in-kind support, developing policy issue briefs, organizing policy workshops, and supporting media education efforts. All national organizations were monitored on 1 overall outcome measure: the percentage of CPPW-funded community objectives that the national organization was matched with that resulted in a policy, systems, or environmental change having been successfully accomplished. CDC provided quarterly data reports on the status of each matched objective, and national organizations were required to submit quarterly progress reports on their activities in support of CPPW-funded communities.

## Results

Three national organizations (American Academy of Pediatrics, American Lung Association, and Society for Public Health Education) were successful in assisting CPPW communities to accomplish 21 (51%) of their 41 tobacco-related matched objectives. In addition, the communities partially accomplished 18 (44%) of their objectives. Some progress was made toward meeting 2 (5%) more objectives at the time data were collected (Table). Examples of matched community objectives included adopting smoke-free policies, supporting hard-hitting counter-advertising, and making cessation services more widely available. Collaborative activities were led by national organizations in support of these matched community objectives (Box 1).

**Table:** National Organizations Support of Community Objectives, Communities Putting Prevention to Work, July 2010–July 2012^a^

Topic/Organization	No. of Matched Communities	Total Matched Community Objectives	Objectives Successfully Accomplished (%)	Objectives Partially Accomplished (%)	Some Progress Toward Meeting Objectives (%)
**Obesity**					
American Heart Association	10	14	12 (86%)	2 (14%)	0
Association of American Indian Physicians	2	7	4 (57%)	3 (43%)	0
BlazeSports America	5	6	6 (100%)	0	0
Community Food Security Coalition	7	8	7 (89%)	1 (11%)	0
National Association of Latino Elected and Appointed Officials Educational Fund	4	12	9 (75%)	3 (25%)	0
National Recreation and Park Association	10	17	14 (82%)	1 (6%)	2 (12%)
Sesame Workshop	6	9	6 (67%)	3 (33%)	0
Totals for obesity prevention	25	73	58 (80%)	13 (17%)	2 (3%)
**Tobacco**					
American Academy of Pediatrics	12	21	15 (71%)	6 (29%)	0
American Lung Association	11	14	6 (43%)	6 (43%)	2 (14%)
Society for Public Health Education	3	6	0	6 (100%)	0
Totals for tobacco use prevention and control	18	41	21 (51%)	18 (44%)	2 (5%)

a Final data as of March 2013. Data were obtained from final evaluation reports of national organizations and from the Centers for Disease Control and Prevention.

Seven national organizations (Association of American Indian Physicians, American Heart Association, BlazeSports America, Community Food Security Coalition, National Association of Latino Elected and Appointed Officials Educational Fund, National Recreation and Park Association, and Sesame Workshop) were successful in assisting CPPW communities to accomplish 58 (80%) of their 73 obesity-related matched objectives. In addition, the communities partially accomplished 13 (17%) of their objectives. Some progress was made toward meeting 2 (3%) more objectives at the time data were collected (Table). Examples of matched community objectives included adopting farm-to-school nutrition policies, improving access to healthful foods through policies and standards, adopting land use policies to increase physical activity, and supporting a physical activity requirement in schools. Collaborative activities were led by national organizations in support of these matched community objectives (Box 1).

During the grant period, national organizations’ technical assistance to matched communities also facilitated policy, systems, and environmental change beyond CPPW-funded communities. Some activities led by national organizations had nationwide impact (Box 2).

## Discussion

In this initiative, national organizations supported local and state health departments in implementing evidence-based interventions by providing education and training, facilitating partnerships, and fostering sustainability. To maximize the effect of the overall CPPW program, the skill sets of specific national organizations were matched with the specific needs of communities. Thus, every community that could be directly supported by a national organization was matched. Given this, there were no prospective community control groups. Nevertheless, in a short time, most matched community tobacco use and obesity prevention objectives were fully accomplished. Successful accomplishment of an objective occurred when community awardees achieved their matched objectives with national organizations in accordance with the timelines for completion for the funding period. Supportive documentation was provided by the awardee that indicated the policy, systems, or environmental change identified in the objective. The qualitative examples provide a snapshot of the substantial policy, systems, and environmental change achieved through these public–private partnerships, both locally and nationally.

In some cases, community awardees did not reach or serve all of the people or settings intended as written in the objective for the funding period, thus only partially accomplishing the objective. For example, 1 objective was stated as follows: “By March 2012, 75% of children in licensed childcare centers in the County will be covered by policies that require 60 minutes of daily physical activity and provide healthy snacks (baseline of 54%).” At the conclusion of the award period, the awardee determined that 72% of children in licensed child care centers were covered, and therefore the objective was marked as partially successful. Although the outcome objective was not met fully, a legitimate case can be made that these are notable achievements as well.

This initiative demonstrated 2 major benefits of public–private partnerships in support of community-based prevention efforts. First, national organizations can accelerate local community action by linking communities to existing national partnerships, acting as facilitators of regional or local partnerships, cultivating community leaders, and creating peer learning experiences among communities. The ability to provide leadership through convening partners and ensuring collaboration is an important role for national organizations (5,6). Second, national organizations support sustainability efforts of communities by developing tools and resources such as curricula and trainings that can be used by communities now and in the future. These organizations can also link communities to local champions and experts in specific areas so that long-lasting relationships can be formed.

Some communities matched with national organizations in this initiative were more likely to fully achieve their objectives, yielding 2 important lessons. One key lesson learned that can increase the effectiveness of similar partnerships in the future is to engage national organizations in supporting community prevention efforts during the planning or early implementation phase. By assessing the needs of the community and determining community readiness at the outset, national organizations are more likely to help communities develop achievable goals and implement an action plan for these goals. A second lesson learned is to more closely align technical assistance options with individual community goals. This includes the development of resources that take into account local laws and policies so that the likelihood of implementation is enhanced.

Through this initiative, expert national nonprofit organizations in tobacco use control and obesity prevention supported policy, systems, and environmental changes at the community level by providing training and education in foundational skills, convening partnerships, and supporting sustainability. Additional public–private partnerships may be warranted to address the nation’s tobacco and obesity epidemics, the major preventable drivers of chronic disease.

**Box 1. Examples of National Organizations and Community Collaborations Involved in Communities Putting Prevention to Work Activities, July 2010–July 2012**

In accordance with US law, no federal funds provided by the US Department of Health and Human Services were permitted to be used by grantees for lobbying or to influence, directly or indirectly, specific pieces of legislation at the federal, state, or local levels. Data were obtained from final evaluation reports of national organizations.

**Obesity prevention**

The American Heart Association developed and implemented a sugar-sweetened beverages education model for improving healthful food procurements with the Boston Public Health Commission. As a result, the Boston Public Health Commission instituted new policies that reduced access to sugar-sweetened beverages and promoted healthful vending options in local health care settings.

BlazeSports America worked with the Mid-Ohio Valley, an area of West Virginia, to secure a daily physical education policy for schools in the region. The Daily Physical Education resolution developed by BlazeSports was adopted or approved in 6 counties and helped to increase the average number of students per school who participate in daily physical education by approximately 23% compared with baseline.

The Community Food Security Coalition supported Miami-Dade County in the implementation of 5 farmers markets. Assistance included development and operations of farmers markets, such as introducing and implementing the use of the Supplemental Nutrition Assistance Program (SNAP) in the markets and outreach to low-income Haitian and Hispanic consumers.

The Miami-Dade Parks Department, in collaboration with the Miami-Dade Health Department and the National Recreation and Park Association, established criteria in accordance with Parks Healthier Vending Guidelines at park and recreation sites throughout the county. As a result, vending machines with healthful items were installed in 25 sites that will serve more than 10,000 children aged 5 to 17 years and other age groups.

The National Association of Latino Elected and Appointed Officials (NALEO) Educational Fund provided technical assistance to the cities of South El Monte and Hawthorne, California (Los Angeles County), to identify and integrate appropriate policies that promote active lifestyles and healthful eating into their community as part of the Healthy Eating Active Living initiative.

**Tobacco use prevention and control**

The American Lung Association provided background information and appropriate messaging to the Rhode Island Department of Health on the sale of flavored tobacco products and on different types of tobacco price promotions and discounts. Subsequently, the Providence City Council passed restrictions on flavored noncigarette tobacco products and on tobacco price promotions and discounts, helping to protect the health of more than 170,000 residents.

The American Academy of Pediatrics shared data, research, resources, and tools specific to smoke-free multi-unit housing with Los Angeles County, leading the city of Compton to pass a smoke-free air law that included public places in multi-unit housing.

**Box 2. Examples of Nationwide Impact of National Organizations Involved in Communities Putting Prevention to Work Activities, July 2010–July 2012**

In accordance with US law, no federal funds provided by the US Department of Health and Human Services were permitted to be used by grantees for lobbying or to influence, directly or indirectly, specific pieces of legislation at the federal, state, or local levels. Data were obtained from final evaluation reports of national organizations.

**Obesity prevention**

The Association of American Indian Physicians (AAIP) provided subcontracts to 10 rural tribal communities across the country, most of which AAIP had not worked with before. The funding allowed the communities to create and maintain community gardens, to create farmers markets to provide rural communities with fresh produce, to increase access to fresh produce at tribal worksites, and to develop safe, accessible areas for physical activity.

The National Association for Family Child Care with technical assistance from the Sesame Workshop revised its accreditation standards for nutrition and physical activity in child care settings. More than 400 state and local family child care provider associations across the United States, which represent more than 1 million family child care providers caring for more than 4 million children, will be affected by these improved standards.

**Tobacco use prevention and control**

The Society for Public Health Education (SOPHE) developed a training program to assist health care providers in initiating and institutionalizing the evidence-based SCRIPT (Smoking Cessation and Reduction in Pregnancy Treatment). SOPHE also developed a cohort of trainers to respond to requests from across the country for training sessions so that SCRIPT can become a part of routine prenatal care.
